# Interpreting the spectral fingerprint of chloroplast movement: implications for understanding photosynthetic light capture and use across scales

**DOI:** 10.1111/nph.70984

**Published:** 2026-02-13

**Authors:** William Woodgate, Troy Magney, Jennifer E. Johnson

**Affiliations:** ^1^ School of the Environment The University of Queensland Brisbane 4072 QLD Australia; ^2^ Terrestrial Ecosystem Research Network The University of Queensland Brisbane 4072 QLD Australia; ^3^ CSIRO Space and Astronomy Kensington 6151 WA Australia; ^4^ Department of Forest Management University of Montana Missoula MT USA; ^5^ Department of Ecology & Evolutionary Biology University of Kansas Lawrence KS 66045 USA

**Keywords:** chloroplast movement, hyperspectral reflectance, leaf optics, nonphotochemical quenching (NPQ), photochemical reflectance index (PRI), plant functional responses, radiative transfer, sun‐induced fluorescence (SIF)

## Abstract

This article is a Commentary on Gan *et al*. (2026), **250**: 845–860.

## Introduction

Remote sensing relies on optical signals (e.g. reflectance, transmittance and sun‐induced fluorescence (SIF)) to infer plant physiological states measured across broad spatiotemporal scales from leaves to ecosystems and seconds to decades. There has been a growing interest in utilising dynamic leaf‐level spectral signals, defined here as operating from seconds to hours, that can provide insight into a myriad of plant functional responses to various resources and stressors (e.g. light intensity and quality, temperature, CO_2_ and humidity; Baránková *et al*., 2016; Magney *et al*., 2017; Van Wittenberghe *et al*., 2021; Pescador‐Dionisio *et al*., 2025). As the leaf‐level spectral signal is an integration of many multi‐scale biochemical processes (Magney, 2025), analysing it requires carefully designed experiments with controls to isolate the causes and consequences of individual processes. In this context, one persistent challenge has been understanding the phenomenon of chloroplast movement (Cm). Although molecular genetic approaches have identified many of the specific molecules mediating Cm, the factors controlling the magnitude of the movements and the physiological consequences of the movements have both remained unresolved. In an article published in this issue of *New Phytologist*, Gan *et al*. (2026; pp. 845–860) cast light on this puzzle with a new technique for measuring Cm and experimental evidence from a phylogenetically broad set of species. Here, we comment on how Gan *et al*.'s work advances quantitative understanding of how Cm fits into leaf structure–function relationships, strengthening the foundation for interpreting leaf spectra and predicting photosynthesis in dynamic environments.

*Overall, Cm alters intra‐leaf light distribution and absorption, which can subtly bias remote sensing signals and warrants further investigation. However, its limited physiological impact suggests it acts more as a fine‐tuning mechanism rather than a dominant driver*.

## Chloroplast movement causes

Plant physiologists have long observed that when light is limiting to photosynthesis, chloroplasts move within leaves to positions that maximise light absorption, whereas when light becomes saturating, they reorient and minimise light absorption. Intuitively, one would expect these rearrangements to enhance the efficiency of photosynthesis under limiting light as well as the efficiency of photoprotection under saturating light – yet leaf‐level physiological studies have not shown clear and consistent support for these predictions. Instead, the experimental evidence suggests that the key to reconciling the role of Cm in leaf structure–function relationships likely lies somewhere in leaf anatomy. It has been well documented that the magnitude of Cm that occurs at any given light intensity varies markedly in relation to leaf anatomy, with stronger movements observed in species with single‐layer mesophyll (e.g. shade‐adapted *Oxalis*) as compared to those with multi‐layer palisade parenchyma (e.g. sun‐adapted *Helianthus*) (Brugnoli & Björkman, 1992). In this article, Gan *et al*. have built on this foundation by developing a new technique to more accurately quantify Cm via evaluation of changes in reflectance and transmittance probed with green light and applying this approach to measure the extent of Cm in a suite of morphologically diverse leaves. The study characterised the quantitative relationship between Cm and anatomical properties of leaf epidermal and mesophyll cells. They found a strong association between the magnitude of Cm and anatomical traits of the epidermis, which focus light on the palisade mesophyll. They also performed experiments demonstrating that elimination of the lens effect, either via application of paraffin oil to the epidermis or abrasion of the epidermis with fine sandpaper, also eliminates Cm. Based on these findings, Gan *et al*. propose that Cm is driven by the light‐focusing lens effect of leaf epidermal cells, which focuses incident light into a higher‐intensity cone that converges within the underlying palisade mesophyll cells. As a corollary, they also propose that the reason why Cm has more modest than expected effects on the efficiency of photosynthesis and photoprotection at the whole‐leaf scale is that the focal plane of the lens effect intersects only a small fraction of the total chloroplast population within leaves. If these conclusions hold across higher plant lineages, they will open a path to explicitly representing Cm within coupled models of leaf optics and photosynthesis – moving the field forward from simply being able to detect this phenomenon, to also being able to make reliable quantitative predictions about its occurrence, magnitude and consequences.

## Chloroplast movement consequences on the photochemical reflectance index and nonphotochemical quenching of fluorescence

The optical and physiological consequences of Cm are subtle but measurable, primarily affecting leaf absorptance, transmittance (Δ*T*/*T* up to > 200% in shade species) and reflectance, with downstream impacts on fluorescence and potential biases in nonphotochemical quenching (NPQ) estimates (Brugnoli & Björkman, 1992; Wilson & Ruban, 2020; Gan *et al*.). Gan *et al*.'s controlled experiments showed small changes (e.g. ≈0.003–0.005 absolute units in reflectance/transmittance at 550 nm) but no significant NPQ alteration when isolating Cm from the lens effect. This raises a key question: to what extent are the numerous NPQ‐related spectral indices, such as the photochemical reflectance index (PRI), confounded by Cm, particularly in natural conditions?

**Fig. 1:**
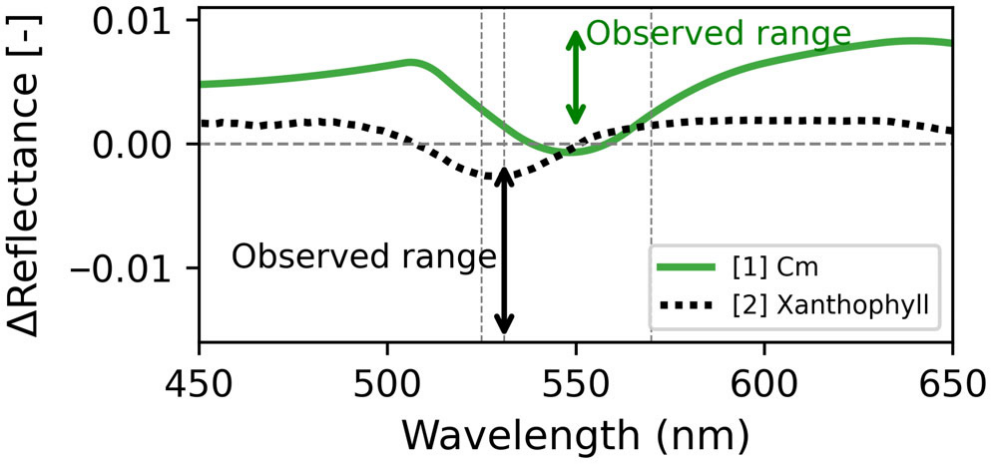
Leaf reflectance difference spectra (light minus dark adaptation in absolute units). Legend interpretation: [1] Chloroplast movement (Cm) from the Arabidopsis *jac1* mutant, adapted from Hermanowicz & Łabuz (2025). [2] Mature *Eucalyptus delegatensis* leaf with de‐epoxidation from the xanthophyll cycle (Lamsal *et al*., 2022). Note the different species used for [1] and [2]. Vertical dashed lines at 525, 531 and 570 nm. The black arrows represent the observed range from NPQ‐related kinetics cited in the commentary. The green arrows represent the range observed in Gan *et al*. (2026; **250**: 845–860, in this issue of *New Phytologist*) at 550 nm caused by Cm.

NPQ is primarily driven by faster biochemical mechanisms (e.g. xanthophyll cycle, ΔpH, PsbS activation and LHCII rearrangement; Demmig‐Adams & Adams Iii, 2006; Ruban, 2016) and slower pigment changes (Porcar‐Castell, 2011; Wong & Gamon, 2015), which do not include Cm as found in Gan *et al*. and Wilson & Ruban (2020). Spectral effects from these NPQ mechanisms can exceed Cm signals by > 10× at the seasonal timescale (Wong & Gamon, 2015). Dynamic PRI‐related changes typically show negative Δ reflectance at 531 nm (magnitudes −0.002 to −0.015 (light : dark adapted) see (Gamon *et al*., 1990; Gamon & Surfus, 1999; Woodgate *et al*., 2019)), opposite in sign to Cm's positive effects (Fig. 1). Hermanowicz & Łabuz (2025) confirm Cm's broad 500–600 nm signature and PRI insensitivity, proposing a Cm‐specific index (CMI) to avoid xanthophyll confounding. The broad range of dynamic spectral effects underscores the importance of observing the full reflectance and transmittance spectrum across timescales to detect Cm.

Overall, under most natural conditions, NPQ‐sensitive indices (i.e. PRI and its variants) are not expected to be substantially confounded by Cm, although the degree of interference depends on the specific formulation and warrants further attention.

## SIF and chloroplast movement

The impact of Cm on SIF is underexplored. Pescador‐Dionisio *et al*. (2025) in leaf‐level experiments recommended dual visible and spectral SIF observations to better characterise NPQ type processes on the SIF signal. In theory, lens‐driven Cm could lead to a higher escape fraction (Fesc) of SIF and higher apparent SIF (and SIF yield), as Cm alters the package effect (self‐shading within chloroplasts) but not the detour effect (light path lengthening), potentially changing reabsorption without affecting true quenching (Wilson & Ruban, 2020). However, PAM‐derived yields like NPQ may be less sensitive to Cm than steady state fluorescence yield (Fs) or SIF due to the maximum fluorescence ($F_{m}^{\prime }$) negligibly changing compared to Fs when NPQ is saturated under moderate light intensities. Hermanowicz & Łabuz (2025) report hyperspectral reflectance shifts (*c*. 2–3% in the 500–650 nm range) from Cm, which could bias SIF estimates if not modelled using process‐based radiative transfer models. In diverse canopies, species‐specific amplitudes may average out, which is tied to palisade width – the dominant explanatory trait for Cm in Gan *et al*. In monocultures like crops, however, Cm could cause much higher SIF variance. This may lead to a minor impact on GPP‐SIF scaling techniques. Future work to further disentangle Cm effects on reflectance, PAM yields and gas exchange metrics with well‐designed experiments is needed (i.e. combining mutants, controls and integrated instrumentation; Magney *et al*., 2017).

## Process‐based models

Experimental work outlined above could be coupled with process‐based model development for enhanced quantification of Cm impacts on spectral signals at leaf and canopy scales. Gan *et al*. demonstrated that leaf surface morphology (epidermal cell shape) is a structural trait influencing internal light dynamics and chloroplast behaviour. Here, convex epidermal cells act like microlenses, focusing light into palisade cells, which triggers chloroplast accumulation in low light. In high light, chloroplasts move to the side walls (avoidance), reducing focused light capture. One approach to capture these effects was presented by Baránková *et al*. (2016), who developed a model called SENELOP to capture deviations from Lambert–Beer's law in leaves caused by Cm. Another approach could be to explicitly represent Cm‐related structural traits in radiative transfer frameworks and test whether their inclusion measurably improves compatibility with dynamic spectral data. For example, trait‐based remote sensing models such as Fluspect‐CX (Vilfan *et al*., 2018), which incorporated the xanthophyll cycle into the leaf‐level Fluspect model, could consider including epidermal cell convexity and palisade cell size as covariates. Any leaf‐level radiative transfer model that can resolve the anatomical features driving Cm can then be coupled to a leaf‐level biochemical model that can resolve the consequences of Cm for photosynthesis and photoprotection (Johnson & Berry, 2021). In our view, this type of model development and evaluation would be best achieved by laboratory and field studies that target the single leaf‐level and leverage high spectral and temporal resolution measurements. The prospects for accurately quantifying Cm are likely to diminish at coarser spectral, spatial and temporal scales.

## Potential chloroplast movement effect at canopy scale

There remains a large challenge in detecting subtle spectral changes like reversible NPQ *c*. 531 nm and Cm at 550 nm at canopy scales. Further, the Cm‐driven changes in 550 nm transmittance and reflectance reported by Gan *et al*. are only on the order of a few percent within a leaf, even in species with relatively large reflectance, whereas canopy‐scale reflectance changes driven by structure and geometry can easily reach order‐of‐magnitude (*c*. 100–200%) differences across view/sun angles and canopy types (Bacour & Bréon, 2005). Additionally, blue‐light phototropin effects are muted in full sunlight vs shade (Takemiya *et al*., 2005), likely further dampening the observable effect from above‐canopy multi‐ or hyperspectral‐sensors where the sunlit signal dominates. Full spectrum hyperspectral observations are increasingly prevalent with UAV and satellite sensor development; however, Cm effects beyond the leaf‐level will quickly diminish with coarser spatial and spectral resolution. An important next step is to test whether species‐level differences in Cm amplitude and kinetics leave any detectable footprint beyond the leaf scale once canopy structure and illumination geometry are accounted for.

## Conclusion

Spectral Cm effects will vary depending on many factors, including light intensity, light quality, light history, species, seasonality, growth and micrometeorological conditions, as well as leaf biochemical composition. A stretch goal for remote sensing enthusiasts could be to integrate Cm amplitude (via green Δ*R* or the Cm index) as a novel functional trait, which could be linked to various plant trait coordination hypotheses across lineages and environments. Overall, Cm alters intra‐leaf light distribution and absorption, which can subtly bias remote sensing signals and warrants further investigation. However, its limited physiological impact suggests it acts more as a fine‐tuning mechanism rather than a dominant driver.

## Disclaimer

The New Phytologist Foundation remains neutral with regard to jurisdictional claims in maps and in any institutional affiliations.
